# Complementary and Alternative Therapies for Chronic Constipation

**DOI:** 10.1155/2015/396396

**Published:** 2015-05-03

**Authors:** Xinjun Wang, Jieyun Yin

**Affiliations:** ^1^Division of Gastroenterology and Hepatology, John Hopkins University, Baltimore, MD 21224, USA; ^2^2nd Clinic Medical School, Nanjing University of Chinese Medicine, Nanjing, Jiangsu 210023, China; ^3^Veterans Research and Education Foundation, VA Medical Center, Oklahoma City, OK 73104, USA

## Abstract

Chronic constipation, an ancient disease, is prevalent, and costly in the general population. Complementary and alternative therapies are frequently used for constipation. This review introduces various methods of complementary and alternative therapies, including acupuncture, moxibustion, massage, and herbal medicine. Efficacy, safety, influence factors, sham control design, and mechanisms of these therapies are discussed and evaluated. Acupuncture or electroacupuncture was found to be most commonly used for constipation among these complementary and alternative therapies, followed by herbal medicine. Although only a small number of clinical studies are flawless, our review of the literature seems to suggest that acupuncture or electroacupuncture and herbal medicine are effective in treating constipation, whereas findings on massage and moxibustion are inconclusive. More well-designed clinical trials are needed to improve and prove the efficacy of the complementary and alternative therapies for constipation; mechanistic studies that would lead to wide spread use and improvement of the methods are also discussed in this review.

## 1. Introduction

Chronic constipation (CC) is a complaining problem for many patients with or without other diseases. The prevalence of constipation in the general adult population ranges from 2% to 26.9%, with a mean of 15.4%, revealed by an integrative literature review of 11 population-based studies. Female gender was identified as the first associated factor in all of these studies, and the second most common associated factor was advanced age [1].

Physical and mental components of quality of life (QoL) scores have been consistently reported to be low in both adult and pediatric patients with CC; meanwhile the greatest influence is seen in secondary care studies [2]. The mean expenditures per hospital costs for constipation increased from $8869 in 1997 to $17,518 in 2010, whereas the total charges increased from $188,109,249 in 1997 to $851,713,263 in 2010 (adjusted for long-term inflation) [3].

The vast majority of CC belongs to functional constipation (FC). According to the Rome III criteria [4], a standardized definition of FC is presented as follows.

*Rome III Functional Constipation Criteria*

1. It must include at least 2 of the following:
  - straining during at least 25% of defecations,
  - lumpy or hard stools in at least 25% of defecations,
  - sensation of incomplete evacuation for at least 25% of defecations,
  - sensation of anorectal obstruction/blockage for at least 25% of defecations,
  - manual manoeuvres to facilitate at least 25% of defecations (e.g., digital evacuation, support of the pelvic floor),
  - fewer than three defecations per week.
2. Loose stools are rarely present without the use of laxatives.
3. There are insufficient criteria for diagnosis of irritable bowel syndrome.

Criteria fulfilled for the previous three months with symptom onset at least 6 months prior to diagnosis.

This definition of FC is for adult patients. For child patients, there are other criteria [4] (as follows).

*Rome III Functional Constipation Criteria*

1. It must include two or more of the following in a child with a developmental age of at least 4 years with insufficient criteria for diagnosis of IBS:
  - two or fewer defecations in the toilet per week,
  - at least one episode of fecal incontinence per week,
  - history of retentive posturing or excessive volitional stool retention,
  - history of painful or hard bowel movements,
  - presence of a large fecal mass in the rectum,
  - history of large diameter stools which may obstruct the toilet.
2. Criteria are fulfilled at least once per week for at least months prior to diagnosis.

CC is very general, including all kinds of constipation, whereas functional constipation is only one major part of it. CC is classified into outlet obstruction constipation (OOC), slow transit constipation (STC), and both. The OOC is characterized with impaired relaxation and coordination of abdominal and pelvic floor muscles during evacuation [5]. STC is defined as prolonged stool transit (>3 days) through the colon [6]. In fact, most of patients with STC are associated with outlet obstruction [7, 8]. It was reported that more than half of patients with STC simultaneously had some degree of outlet obstruction [9, 10].

Pharmacologic agents for CC are available. However, 28% of participants were dissatisfied with their laxatives. In a large sample survey, as high as 83% of respondents indicated that they were absolutely or probably interested in other treatment options and complementary/alternative therapies [11]. In another survey, Johanson and Kralstein reported that the causes of laxatives dissatisfaction included “does not work well” or “inconsistent results” and safety-related or adverse-effect concerns [12]. In children, the adherence rate to medical therapies of constipation was reported to be low, attributed to financial difficulties (23.2% of cases) and side effects (40.2%) [13].

This article reviews complementary and alternative therapies for CC, including acupuncture, moxibustion, massage and herbal medicine.

## 2. Acupuncture

Acupuncture is an ancient Chinese Traditional Medicine therapy in which acupoints on skin are manually stimulated by needles. It is usually termed hand-acupuncture. Electroacupuncture (EA) is a method in which electrical current is delivered to needles inserted into acupoints. Transcutaneous electroacupuncture (TEA) is similar to EA but the needles are replaced with electrodes. Auricular acupuncture (AA) is the one in which acupuncture is performed at acupoints on the skin of ear. All of the above methods had been used in the treatment of CC.

Clinic studies on acupuncture or EA for CC were searched in PubMed database from inception to October 2014. Keywords used in the search included “acupuncture” or “electroacupuncture” and “constipation”. The language of publications was instructed as English or abstract in English. Seventeen reports yielded from this search were summarized in Table 1.

### 2.1. Quality Assessment of Acupuncture Trials for CC

Among the 17 articles, 11 of them were RCT's and 90% of the RCT studies were published after 2010. There were 6 high quality trials [14, 16, 17, 23, 24, 27] which could be assessed as 5 according to Jadad scoring system [31], but sample sizes of them were all small. A trial containing 553 samples was evaluated to have a Jadad score of less than 3 due to the flaw in design [15].

Multiple methods of the design for control were used in clinical studies on CC. The control groups in the literature included medications, other methods of stimulation, and acupuncture plus medications. Medications used in the control group included conventional medicine [15, 16, 23, 24] (Mosapride, Macrogol 4000, Lactulose) and Chinese herbal medicine [15, 22] (Fuzhengliqi mixture and Plantain and Senna Granule). Sham acupuncture [17, 27, 29], shallow acupuncture [16, 23, 24], regular electrical stimulation [19], and other methods of stimulation were performed as control methods. Combinational use of medications included EA plus Fuzhengliqi mixture [15], and EA plus Plantain and Senna Granule [22]. There was only one trial in which two kinds of stimulation methods, acupuncture and moxibustion, were used together [21].

The treatment duration [14–17, 22–24, 27] ranged from 4 weeks to 7 weeks, and the follow-up time [14–16, 22, 24, 26, 27] ranged from 4 weeks to 64 weeks. The primary outcome was the number of weekly spontaneous bowel movements. The secondary outcomes included opaque X-ray marker, patient's satisfaction, and clinical symptom score (such as weekly defecation frequency, defecation time, stool characteristics, straining and abdominal pain). The questionnaires used in trials included Bristol score, Cleveland Clinic Score, and Quality of Life. Some indicators about mechanisms of acupuncture for constipation also were measured, including plasma motilin [15], plasma panopioid [29], and heart rate variability [17].

### 2.2. Efficacy and Safety of Acupuncture for CC

Several systematic reviews, including meta-analysis, indicated that acupuncture for CC was effective and did not cause obvious adverse events [32–36].

The overall efficacy rate of hand-acupuncture for chronic functional constipation was 52.0% [21]. It improved weekly spontaneous defecation times, abdominal pain, evacuation difficulty, endless sensation of defecation, obstruction sense of anus, laxative prescription dependence, and quality of life [21, 32], as well as psychological symptoms score [21].

The overall efficacy rate of EA for chronic functional constipation raged from 54.6 to 94.4% [15, 19, 27]. EA increased the frequency of weekly defecation and the number of persons who had defecation 4 times or more a week (responder) [16, 23], decreased stool property, constipation symptom grade, accompanying symptom grade, and gastrointestinal transit time (GITT) [15, 22, 24, 27].

Several articles reported that acupuncture or EA outperformed conventional medicine, such as lactulose [16, 23, 24] and Plantain and Senna Granule [22]. This was different with the conclusion drawn from a systematic review which indicated that acupuncture was probably as effective as conventional medical therapy in the change of bowel movements and colonic transit activity [32]. This difference might be attributed to the small sample sizes in these trials. A trial including 553 patients reported that the effective rate of EA was not different from Fuzhengliqi mixture or Mosapride combined with Macrogol 4000 in short term but was superior to them in long term because constipation symptoms recurred in the two control groups [15].

Zhou et al. performed an RCT study and reported that the effective rate of AA for functional constipation was 92% [18]. However, the reliability of this conclusion was low due to small sample size and lack of control. It was indicated in a systematic review that no conclusion should be made on the effectiveness of acupuncture due to significant methodological flaws [34].

Acupuncture for the treatment of pediatric patients with hospital-induced constipation was evaluated in a pilot study for the feasibility and acceptability with encouraging results [20].

### 2.3. Most Popular Acupoints for CC

Acupoints used more than 3 times for CC in the 17 articles included ST25, ST37, BL25, ST36, TE6, CV6, CV4, BL33, and BL34 (Table 2). These acupoints usually are considered as representative choices adopted by doctors and researchers. The top five acupoints being used most frequently for treating CC are discussed here.

ST25 is on the upper abdomen, laterally to the umbilicus above the small intestine according to World Health Organization (WHO) standard acupoint locations [37]. EA at ST25 was reported to enhance small intestinal motility in rodent model of slow transit constipation [38]. However, in normal or fasted rats, EA at ST25 was found to produce inhibitory effects on jejunum electrical and mechanical activities [39, 40]. These findings seem to suggest that EA at ST25 exerts different effects under different conditions.

ST36 and ST37 are located on the anterior aspect of the leg and above of tibialis anterior muscle. ST36 is above ST37 [37]. Acupuncture stimulation of ST36 was reported to increase intragastric pressure and gastric peristaltic frequency in rats with gastric hypomotility [41]. In patients after abdominal surgery, ST36 was able to shorten the time of first flatus passage and improve gastrointestinal functions [42]. Significant acceleration of colonic transit with EA at ST36 was mediated via the sacral parasympathetic efferent pathway [43]. Acupuncture at ST37 was reported to alter rectal motility and the effect appeared one hour after needling [44].

BL25 is located on the lumbar region, at the same level as the inferior border of the spinous process of the fourth lumbar vertebra (L4), laterally to the posterior median line [37]. Acupuncture at BL25 reduced early postoperative inflammatory small bowel obstruction [45], improved symptoms of ulcerative colitis [46], and irritable bowel syndrome [47].

TE6 is located on the posterior aspect of the forearm, midpoint of the interosseous space between the radius and the ulna, proximal to the dorsal wrist crease [37]. EA at TE6 and ST36 was effective for adhesive ileus, remarkably improved abdominal pain and distention, and accelerated intestinal peristalsis [48].

The above discussion indicates that acupuncture or EA at all of the top five acupoints improves gastrointestinal motility. According to the anatomy of the nervous system, tibial nerve, L4 spinal nerve, and posterior interosseous nerve are under ST36 and ST37, BL25, and TE6, respectively. Therefore, acupuncture effects of these four acupoints are probably mediated via these nervous pathways. Special acupuncture technique is required on ST25 to get a better therapeutic effect. In this technique, the needle is inserted perpendicularly and slowly till penetrating the peritoneum, about 20–65 mm in depth [16]; direct intestinal stimulation might be implicated with this technique.

### 2.4. Influence Factors of Acupuncture for Constipation

There are several factors influencing the effective rate of acupuncture for CC [21, 27]. These include acupoint group, operative technique of puncture, stimulation parameters, and treatment interval.

Various acupoint groups had been used in clinical trials. All of acupoints for CC can be classified into four categories according to their locations: abdomen acupoints (ST25, ST28, CV4, CV6, SP15), lumbosacral acupoints (BL25, BL20, BL23, BL33, BL34), crus acupoints (ST36, ST37, BL57, SP6), and forearm acupoints (TE6, LI11, LI4). Acupoint groups result in the combination coming from at least one kind of acupoints. Abdomen acupoints plus crus acupoints or forearm acupoints are counted as acupoint group regularly [14, 17, 19, 21, 22, 25, 26, 28, 30]. Lumbosacral acupoints are taken as a group usually [21, 25]. One trial used three kinds of acupoints simultaneously: abdomen, lumbosacral, and crus [15]. In five trials, only one acupoint was used [16, 20, 23, 24, 27]. No studies are available in the literature comparing different acupoint groups. Studies of searching optimal acupoint group are needed.

ST25, the most frequently used acupoint, was dealt with through a special operative technique of puncture which was named as deep-puncture technique [16, 23, 24]. Here is the deep-puncture technique of ST25: needle is inserted perpendicularly and slowly till penetrating the peritoneum, about 20–65 mm in depth [16]. Using the deep-acupuncture technique, the number of functional constipation patients whose defecation was up to 4 times per week was increased, compared with the shallow-acupuncture technique during the second treatment week [23]. However, at the forth treatment week there was no difference between the two techniques in the number of responders, the defecation interval, stool property, constipation symptom grade, accompanying symptom grade, or GITT [16, 23]. At the 6-month follow-up, deep-acupuncture was reported to be still effective, whereas the shallow-acupuncture became ineffective [24]. The standard definition and operation about “deep-acupuncture” of ST25 was studied in the fields of anatomy and safety [49]. In acupuncture theory, the operative technique of puncture is considered as one of key factors that affects the outcomes of acupuncture. Therefore, the direction and depth of needling are required. This technique was applied in puncturing ST25 for constipation, but not for other acupoints and other diseases.

There are 11 trials which adopted EA for constipation among the 17 articles. The parameters used in EA treatment seem to be important. Different stimulation frequencies were used in these studies, including 2 Hz/200 Hz [15], 1 Hz/20 Hz [19], 2 Hz/100 Hz [22], and 10 Hz [30]. In rough, EA frequency can be divided into low-frequency (1 Hz, 2 Hz, 10 Hz, etc.) and high-frequency (100 Hz, 200 Hz, etc.). In acupuncture analgesia, high- and low-frequency of EA could facilitate the release of endogenous opioid peptides. The effect of low-frequency EA was found to be mediated by the *κ* opioid receptor, whereas high-frequency EA was reported to be mediated by the *δ* and *μ* opioid receptors [50]. However, it is unclear whether the EA frequencies for analgesia are applicable to EA for constipation and more studies are needed to determine the best EA stimulation frequency for constipation.

In addition to the stimulation frequency, the frequency of treatment (treatments per week) is also an important factor. Five treatments per week seemed to be most popular in the previous studies [15, 16, 22–24]. Most of acupuncturists believe that efficacy induced by acupuncture can be maintained for one or two days and thus require patients to receive treatment every day or every other day. However, one of major problems with clinical acupuncture is that the treatment is administrated infrequently, such as 1 or 2 times per week, yielding insignificant or inconsistent results [30].

### 2.5. Sham Acupuncture Design

Sham acupuncture was used as control in two of the articles [17, 27]. Sham acupuncture design is based on two key points: one is the use of nonacupoints and the other is nonneedle. For blindfolding patients, sham needles were glued on skin. It looks like being inserted; however, this is exposed easily for experienced patients due to different feelings between the needle being inserted at the acupoint and the one placed on the surface of acupoint. Sham acupuncture at nonacupoints refers to needle manipulation at points that are not on any meridian or acupoints. Different from the specific technique of acupuncture, which can induce a higher intensity of* de qi* that substantially improves the therapeutic effect, acupuncture that does not induce* de qi* can also be regarded as sham acupuncture. This method of sham design was used in acupuncture for Bell's palsy, a recent RCT completed by Xu et al. [51], and appreciated by John Fletcher who is Editor-in-Chief of Canadian Medical Association Journal. Fletcher considered that results of that trial were reasonable because every patients received acupuncture, but with valid or invalid technique [52]. What calls for special attention is that valid or invalid technique should be defined according to different diseases and types of acupuncture. For example, EA-shallow being regarded as control in some trials [16, 23, 24] should not be designed as sham control, unless electric current was shut off.

### 2.6. Mechanisms of Acupuncture for Constipation

Despite the fact that acupuncture for constipation has been proved effective in clinical studies [32], enhancing contractility in the distal colon [53], and accelerating colonic transit [43] in animal studies, mechanisms involved in these effects are still unclear. A lower level of motilin was noted in patients of functional constipation and found to be elevated with acupuncture at ST36 and ST37 [54]. EA at bilateral ST25 was reported to increase colonic smooth muscle thickness and number of Cajal cells considerably [38]. Vagal and parasympathetic mechanisms have also been implicated in the accelerative effect of acupuncture or EA on colon motility [55]. Overall, little is known on the mechanisms involved in the effect of acupuncture on constipation. More studies are needed to reveal possible pathways, such as neural pathway, endocrine pathway, opioid pathway, and/or serotonic pathway.

## 3. Moxibustion

Moxibustion is a traditional therapy in Chinese Medicine to stimulate acupoints with burning moxa made from dried mugwort. Little has been reported in the literatures on the management of CC with moxibustion. A systematic review [56], published in 2010, only included 3 RCTs with a total of 256 patients, and no randomization or blinding (two in Chinese and one in Korean). Given that the methodological quality of these trials was poor, the review reported that there was insufficient evidence to suggest that moxibustion was an effective treatment for constipation [56].

In PubMed database, RCTs of moxibustion for CC were searched from its inception to October 2014 with keywords including “constipation” plus “moxibustion,” resulting in only one high quality RCT published in 2011 in English. This trial was randomized, sham-controlled, patient blinded, and pilot clinical [57]. The trial noted that moxibustion treatment appeared safe but showed no positive effect on constipation [57].

However, this conclusion does not stand up to be scrutinized due to the design of sham control. Sham moxibustion used in this trial [57] was given with adding insulation below the moxa pillar in order to prevent the transfer of heat from patients. The sham moxa pillar looked similar to real moxa pillar on its appearance and burning procedure and that the smoke from moxa could be smelled and the burning could be observed. This method of sham moxibustion seems well established as blinded to the participants [58, 59]. However, sham moxibustion would be recognized easily by experienced patients and thus patients with previous experience of moxibustion should be excluded from a controlled study [59].

Studies of moxibustion for constipation have been so limited that no mechanistic research has been published. Long-term, larger sample size, rigorously designed, and mechanism studies are desired.

## 4. Massage

Massage is the manipulation of activating deeper and superficial layers of connective tissues and muscles using various techniques. It has been practiced for thousands of years in many ancient civilizations [60].

Seventeen clinic articles were derived from the PubMed search with keywords “massage” and “constipation” [61–77]. Among them, there are only 3 articles with a Jadad score ≥3 [31]. In spite of this, the 3 articles were in lack of sham control and blind method and of very small sample size. In brief, these 17 studies showed that massage increased defecation frequency [63, 65, 66, 76], relieved abdominal pain syndrome [66], and decreased Gastrointestinal Symptoms Rating Scale [66] and Constipation Assessment Scale [71] but could not decrease laxative use [66].

Various mediums have been used in manipulation of massage, but it is unclear which methods are better. Aroma oil, which is often used in massage, did not seem to be more effective than the meridian massage [65]. Massage may work on constipation in children and seniors. A study indicated that massage was beneficial to hospitalized children with constipation due to brain injury [61]. But it is not recommended because of the lack of sufficient evidence according to an integrative review of the literature [78]. Abdominal massage using essential oils seems helpful for constipation in the elderly [71].

It is difficult for massage to design a method of sham or blind technique. Various techniques of massage have been developed through thousands of years originated from different ancient civilizations. Up till now there is no well-recognized standard technique for massage. Therefore, technique of sham or blind massage could not be defined.

Abdominal massage was performed in patients with constipation and healthy volunteers with negative results. Neither in patients, nor in healthy controls, did the abdominal massage alter stool frequency or colon transit measured by radiopaque markers [75].

There are a number of advantages with massage. Firstly, despite the fact that the trials about massage for constipation were various in terms of designs, patient samples, and types of massage used, there were no adverse side effects. Secondly, massage can be self-administrated or administrated by patients since it is easily learnt [77]. Thirdly, expenditure and cost-effectiveness could be reduced greatly since it can be self-administrated [79].

Overall, the experience of abdominal massage is appreciated by consumers, not only feeling embraced and in safe hands but also improving their bowel habits [62].

## 5. Herbal Medicine

Constipation, as an ancient disease, has been treated with many kinds of herbal medicines in the human history. According to quantity of herbal medicines, it can be divided into two types: single herb and multiple herbs. According to active ingredient of single herb, it also can be divided into two types: bulk laxative and stimulant laxative.

### 5.1. Single Herb Medicine

#### 5.1.1. Bulk Herbal Laxative

Psyllium and* Ficus carica* are frequently used bulk laxatives. Psyllium increased stool frequency and improved stool consistency but was not effective on colon transit or anorectal motility [80]. Similar results were reported in CC patients with Parkinson's disease [81]. Psyllium increased more stool water content and weight, more total stool output, and higher O'Brien rank-type scores than docusate sodium according to a multicenter, randomized, double-blind, and parallel-design study in which 170 subjects with chronic idiopathic constipation were treated for 2 weeks [82]. About the efficacy of Psyllium for constipation, a general understanding is that its high fiber and mucilaginous content contribute to a laxative action. Gut-stimulatory effect of Psyllium, mediated partially by 5-HT_4_ (5-hydroxytryptamine 4) receptor and muscarinic receptor activation, was beneficial as complement actor [83]. However, high dose Psyllium was effective on diarrhea resulting from its inhibitory effect on the gut possibly mediated by activation of nitric oxide-cyclic guanosine monophosphate pathways and blockade of Ca^2+^ channels [83].

*Ficus carica* was not used in clinic trials despite the fact that it is considered as laxative in some countries.* Ficus carica* paste for loperamide-induced constipation in rats increased pellet number, weight, water content, tension, and peristalsis of intestinal ileum, as well as thickness and mucin area in the distal colon [84]. No abnormal symptoms were observed on serum and whole blood parameters [84]. Similar results were obtained in constipated beagles induced by a high-protein diet and movement restriction [85]. The ameliorating effect on constipation was believed to be attributed to cellulose, one of the main components of* Ficus carica* [84, 85]. Cellulose improved fecal excretion by increasing water content and bulk, elevating viscosity and shortening fecal transit time [86].

#### 5.1.2. Stimulant Herbal Laxative

Anthranoid-containing laxatives, the most frequent in stimulant herbal laxatives, include senna, aloe, rheum officinale, and cascara.

Anthraquinones are effective components in this kind of stimulant herbal laxatives. Glycosides, naturally occurring from senna, aloe, rheum officinale, and cascara, pass unchangedly through the small intestine and are split into active ingredient rhein-anthrone by the colonic microbiota [87]. They were reported to improve stool frequency and consistency in a number of clinical studies [88–90]. Pseudomelanosis coli or melanosis coli which are a dark-brown discoloration of colon mucosa would be induced by anthraquinone in 9–12 months [91] and would disappear over weeks to months after termination of the use of anthraquinone [92]. It is controversial whether there is a link between pseudomelanosis coli and colorectal cancer.

### 5.2. Multiple Herbs Medicine

Multiple herbs medicine means two or more of single herb medicines are used in combination. For example, Psyllium and senna as a group occurs in a lot of over-the-counter brands. Agiolax, a representative sample, comprising* Plantago ovata* 52 g, ispaghula husk 2.2 g, and* Tinnevelly senna* Pods 12.4 g per 100 g granules, was proved superior to lactulose in measurement of mean daily bowel frequency, stool consistency, and ease of evacuation in a double-blind crossover study [93]. The expansion of Psyllium and stimulation of sennosides under safe and recommended doses are perfect in cooperation.

### 5.3. Chinese Herbal Medicine

Chinese herbal medicine for constipation is complex on its formation. Usually, it comprises not only multiple herbal laxatives but also some other herbs which contribute to relieve side effect of stimulant herbal laxatives, for example, Ma Zi Ren Pill [94–96] and CCH1 [97].

Ma Zi Ren Pill, who's other name is Hemp Seed Pill, comprises six herbs: Semen Cannabis Sativae, Radix Paeoniae, Semen Pruni Armeniacae, Fructus Immaturus Citri Aurantii, Radix et Rhizoma Rhei, and Cortex Magnoliae. According to the Chinese traditional medicine theory, it moistens the intestines, relaxes the bowel, and promotes the movement of Qi [95]. An 18-week prospective, randomized, double-blind, placebo-controlled clinical study on 120 subjects documented that Ma Zi Ren Pill increased complete spontaneous bowel movement and decreased straining at evacuation and no serious adverse effects were noted [95].

CCH1 comprises six herbs:* Panax* ginseng C. A. Meyer, Zingiber officinale Rosc.,* Glycyrrhiza uralensis* Fisch.,* Atractylodes macrocephala* Koide,* Aconitum carmichaelii* Debx., and Rheum tanguticum Maxim [97]. A randomized, double-dummy, double-blind, and placebo-controlled trial on 120 participants showed that CCH1 was superior to lactulose in spontaneous bowel movements [97]. Another high quality trial showed that efficacy of CCH1 could be proved, but maintenance effect needs further trial [98].

The two Chinese herbal medicines were tested in high quality trials. However, the evidence and reliability of many others are compromised by methodological flaws [99]. Further randomized, placebo-controlled, double-blind trials need to be promoted and reported in detail [99].

## 6. Conclusion

Among the four kinds of complementary and alternative therapies for constipation discussed in this review, the efficacy of acupuncture and herbal medicine has been indicated. Well-designed high quality studies are needed to investigate the efficacy of moxibustion and massage for constipation. Since constipation is a chronic and highly prevalent disease, convenient and cost-effective therapies are needed. Therefore, complementary and alternative medicine is expected to play a more important role in the future. Novel and innovative therapies of complementary and alternative medicine are needed in treating constipation. To increase the efficacy of existing methods, combinational methods may be explored. Equally, if not more importantly, mechanistic studies are needed in order to improve and disseminate the application of the available complementary and alternative therapies for constipation.

## Figures and Tables

**Table 1:** Articles of acupuncture or EA for CC.

Reference	Study design (participants)	Acupoints	Implementation of acupuncture	Key efficacy results	Adverse reactions
Wu et al., 2014 [14]	RCT (*n* = 104) adult	ST25, BL25, LI11, ST37	EA1: ST25, BL25 EA2: LI11, ST37 EA3: ST25, BL25, LI11, ST37 C: Mosapride citrate	Weekly frequency of defecation, defecation difficulty life, and quality score were all improved significantly in the four groups; in follow-up, weekly frequency of defecation of LI11 and ST37 (EA2) was superior to the other three groups	NA

Zhang et al., 2013 [15]	RCT (*n* = 553) adult	ST25, ST37, ST36, BL25, TE6	EA: 2 Hz/200 Hz D: Fuzhengliqi mixture EA + D: both of above C: Mosapride and Macrogol 4000	All groups decreased the defecation interval, stool property, constipation symptom grade, accompanying symptom grade, and GITT; EA + D was better than others; EA could keep long-term effect	No

Peng et al., 2013 [16]	RCT (*n* = 128) adult	ST25	EA-deep: 20 to 65 mm in depth EA-shallow: 5–8 mm depth D: lactulose oral liquid	All groups increased the weekly defecation frequency; EA-deep could keep long-term effect	No

Chen et al., 2013 [17]	RCT (*n* = NA) adult female	ST36, ST37, ST25, ST28, CV4, CV6	EA Sham-EA	EA improved constipation symptoms and increased autonomic nervous system activities, sham-EA not	NA

Zhou et al., 2012 [18]	RCT (*n* = 200) elder	AT3, 4i, AT3, AT4, CO7, CO17, AH8, CO18, Constipation Point	AT: according to the pattern/syndrome differentiation C: solid points	The effective rate: AT 92.0%, C 76.0%	NA

Xu et al., 2012 [19]	RCT (*n* = 64) adult	TE6, ST25, ST36, ST37	EA: Hwato neuro and muscle stimulator C: regular electronic stimulator	The effective rate of short term: EA 54.6%, C 29.0%	NA

Anders et al., 2012 [20]	Retrospective case series study (*n* = 10) children	Quchi (LI11)	Fixed indwelling acupuncture needles (0.9 mm in length)	After a median of 3 days of HIC, all children defecated within 2 h. Local constipation therapy was not required	No

L.-J. Wang and L.-L. Wang, 2011 [21]	RCT (*n* = 100) adult	Group 1: ST25, SP15, CV6, CV4, ST36, ST37, SP6. Group 2: BL33, BL34, BL5, BL23, BL20 Alternatively	HA: punctured by hands HA + moxibustion: grain-shaped moxibustion was given at CV6, ST36, BL25, BL20, and others with puncture	The total effective rate: HA + moxibustion as 74.0% (37/50) versus 52.0% (26/50)	NA

Guo et al., 2011 [22]	RCT (*n* = 378) adult	ST25, ST37, ST36, BL25, TE6	EA: 2 Hz/100 Hz D: Plantain and Senna Granule EA + D: both of the above	All groups decreased the scores of defecation cycle, stool property, constipation symptom grade, accompanying symptom grade, and GITT; EA + D was better than others; EA and EA + D could keep long-term effect	No

Wang et al., 2010 [23]	RCT (*n* = 95) adult	ST25	EA-deep: 45 mm in depth EA-shallow: 5 mm in depth D: lactulose oral liquid	EA-deep and EA-shadow were significantly superior to D group in increasing number up to 4 and improved CCS. EA-deep worked faster than EA-shadow	NA

Wang et al., 2010 [24]	RCT (*n* = 95) adult	ST25	EA-deep EA-shallow D: Duphalac	EA-deep was similar to EA-shallow in number up to 4 and CCS, and its efficacy remained much longer	NA

Jin et al., 2010 [25]	Before-after study (*n* = 90) adult	Group 1: ST25, CV6, ST37; Group 2: BL33, BL34, BL25 Alternatively	EA: BL33, BL34, ST25, T37	The scores of defecation frequency, difficulty degree of defecation, defecation time, endless sensation of defecation, stool quality, awareness of defecation, and QoL were obviously improved after treatment. The total effective rate was 67.7% (61/90)	NA

Ding et al., 2009 [26]	Before-after study (*n* = 30) adult	Group 1: ST25, SP15, SP14, CV6, CV4, ST36, ST37; Group 2: BL25, BL23, BL31, BL32, BL33, BL34, Ex-HN1 Alternatively	Deep needling was applied on acupoints of abdominal and back region and moxibustion was put on Ex-HN1	Reduced laxative, scores for awareness, and QoL. Increased frequency of defecation	No

Zhang et al., 2007 [27]	RCT EA SA	TE6	EA: EA at Zhigou SA: EA at nonacupoint	EA could obviously improve CCS and CTT, decrease cathartics, effective rate of 94.4%	No

Zhu et al., 2003 [28]	Before-after study (*n* = 188) adult	ST25, ST36, ST37, BL25, BL57	HA	Total effective rate of 100%	NA

Broide et al., 2001 [29]	CCT-self (*n* = 17) child	NA	Treated by five weekly placebo acupuncture sessions, followed by 10 weekly true acupuncture sessions	The frequency of bowel movements increased only after 10 true acupuncture sessions	NA

Klauser et al., 1993 [30]	CCT-self (*n* = 8) adult	LI4, ST25, LE3, BL25	EA: 10 Hz	Stool frequencies and CCT were not altered	Two patients dropped out because symptoms worsened

RCT: randomized controlled trial; CCT: controlled clinical trial; HA: hand-acupuncture; EA: EA; AT: auriculotherapy; SA: sham acupuncture; D: drug; HA + D: hand-acupuncture + drug; EA + D: EA + drug; C: control; PE: patient's endurance; MA: mean age; PO: by mouth; CCS: Cleveland Constipation Score; number up to 4: the number of constipation patients whose defecation was up to 4 times per week; BMs, bowel movements; GITT: gastrointestinal transit time; TGITT: total gastrointestinal transit time; M-ITT: mouth-intestine transit time, CTT: colonic transit time; RCTT: right colonic transit time; LCTT: left colonic transit time; RSTT: rectosigmoid colonic transit time; MTL: motilin; QoL: quality of life; CI: confidence interval; QD, every day; BID: twice per day; TID: triple per day; NA: not acquirable.

**Table 2:** Acupoints appeared ≥3 times for CC in these 17 articles.

Acupoints	Times appeared
Tianshu (ST25)	13
Shangjuxu (ST37)	9
Dachangshu (BL25)	8
Zusanli (ST36)	7
Zhigou (TE6)	5
Qihai (CV6)	4
Guanyuan (CV4)	3
Zhongliao (BL33)	3
Xialiao (BL34)	3
